# Developmental and Epileptic Encephalopathy Due to a Novel *ARHGEF9* Deletion Variant: Case Series of Two Siblings

**DOI:** 10.31083/RN46598

**Published:** 2026-05-26

**Authors:** Jie Li, Guangshun Han, Yizhi Wei

**Affiliations:** ^1^Department of Neurology, Liuzhou People's Hospital Affiliated to Guangxi Medical University, Liuzhou Key Laboratory of Epilepsy Prevention and Research, 545000 Liuzhou, Guangxi, China

**Keywords:** *ARHGEF9*, epilepsy, gene deletion, intellectual disability

## Abstract

**Introduction:**

Developmental and epileptic encephalopathy (DEE) is a group of severe neurological disorders characterized by early-onset epilepsy and developmental delay, often caused by genetic variants. Cdc42 Guanine Nucleotide Exchange Factor 9 (*ARHGEF9*) gene variants have been linked to DEE, yet novel variants and their phenotypic presentations remain incompletely characterized.

**Clinical Cases:**

Herein, we describe two siblings with DEE caused by a novel deletion variant in the *ARHGEF9* gene. Both patients presented with early-onset epilepsy and developmental delay. Whole-exome sequencing identified a hemizygous c.1037_1045del variant in the *ARHGEF9* gene (NM_015185.2) in both brothers, which is reported here for the first time. Notably, the two siblings exhibited a marked difference in outcomes: the elder brother achieved good seizure control with anti-epileptic drugs, while the proband, despite multidrug therapy and vagus nerve stimulation (VNS), exhibited a limited response and continued to experience frequent seizures.

**Conclusions:**

These cases expand the genotypic spectrum of *ARHGEF9*-related disorders and underscore the intrafamilial phenotypic variability associated with this gene. These findings emphasize the significance of early genetic testing for establishing a diagnosis, assessing prognosis, and facilitating genetic counseling.

## 1. Introduction

Developmental and epileptic encephalopathy (DEE) encompasses a group of disorders characterized by developmental impairment accompanied by frequent epileptic activity, resulting in intellectual and motor regression as well as developmental delay. In children, the primary clinical manifestations of DEE include early-onset epilepsy, developmental delay or regression, and abnormal electroencephalogram (EEG) findings. The etiology of DEE is complex, with genetic factors accounting for more than half of the cases [1]. Among these genetic causes, DEE8 (OMIM #300607) constitutes a distinct X-linked disorder attributed to pathogenic variants in the Cdc42 Guanine Nucleotide Exchange Factor 9 (*ARHGEF9*) gene [2]. This article describes the clinical features, biochemical tests, EEG, cranial magnetic resonance imaging (MRI), and genetic findings of two patients with DEE associated with variants in the *ARHGEF9* gene to enhance clinicians’ understanding of this gene and its associated disorders.

## 2. Case Report

This case series is reported in accordance with the CARE guidelines checklist (see **Supplementary Material**). Patient 1 (proband): A 15-year-old male patient was admitted with a chief complaint of “episodic limb convulsions for 14 years”. His medical history was as follows. At the age of 11 months, he began experiencing episodes characterized by paroxysmal ocular deviation (either left or right), occasionally accompanied by unilateral or bilateral limb twitching. These episodes lasted 1–2 minutes and occurred 1–3 times per month. Initial evaluations, including local medical consultations, computed tomography (CT), and video electroencephalography (VEEG), revealed no significant abnormalities. Furthermore, both blood tandem mass spectrometry and urine organic acid analysis showed no abnormalities, effectively ruling out metabolic genetic disorders. The patient was initially treated with topiramate, which resulted in a gradual reduction in seizure frequency, leading to a seizure-free period of approximately two years. However, at approximately 3 years of age, seizures recurred, characterized by unresponsiveness, limb stiffening, and shaking, with episodes resolving within approximately 1 minute. Subsequent treatment included the gradual addition of medications such as levetiracetam, lamotrigine and phenobarbital, which resulted in some improvement. Nevertheless, intermittent seizures persisted at variable frequencies, ranging from once a month to several times per month. The current seizure types observed in the patient are as follows: (1) Focal onset aware seizure, manifesting as episodic eye deviation to the left or right, occasionally accompanied by unilateral or bilateral limb twitching. (2) Generalized onset tonic-clonic seizure. Patient 2: The proband’s 17-year-old elder brother. At 18 months of age, he developed unprovoked episodes of right-sided or secondarily generalized limb convulsions, each lasting approximately 1 minute, with a frequency of 3–5 episodes per year. Sodium valproate was initiated, achieving seizure freedom for about 5 years. Seizures subsequently recurred with similar semiology at about 8 years of age, with a frequency of 1–3 times per month. Following the addition of oxcarbazepine, the seizure frequency decreased to 1–2 episodes per year. Currently, his seizure types consist of focal aware seizures, manifesting as right-sided limb convulsions that occasionally progress to bilateral tonic-clonic seizures.

**Fig. 1.**
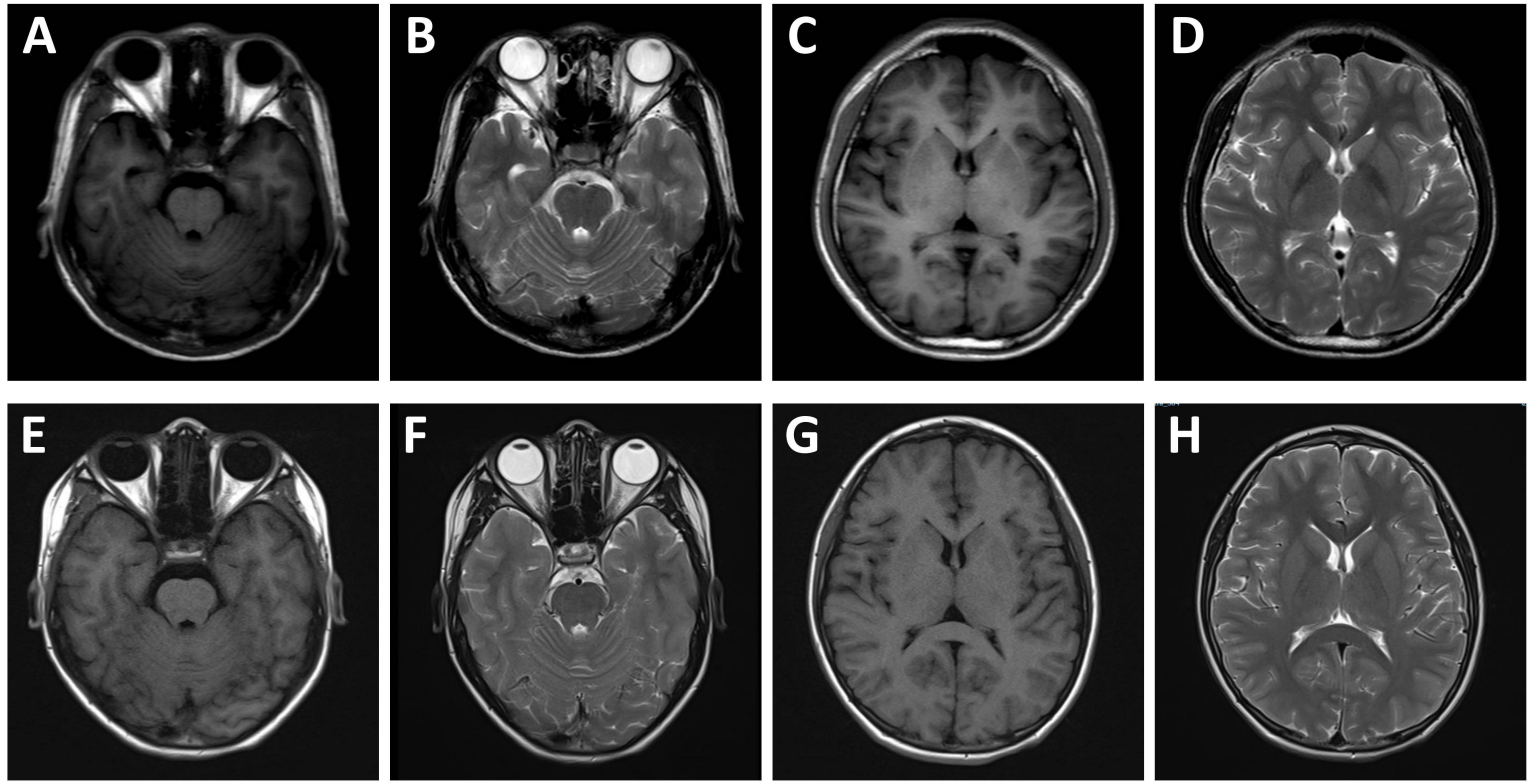
**Cranial magnetic resonance imaging (MRI) findings of the patient and his elder brother**. MRI of the patient (A–D) and his elder brother (E–H). The cerebral hemispheres appear symmetrical, with a clear demarcation between gray and white matter, and no abnormal signals are observed within the brain parenchyma.

**Fig. 2.**
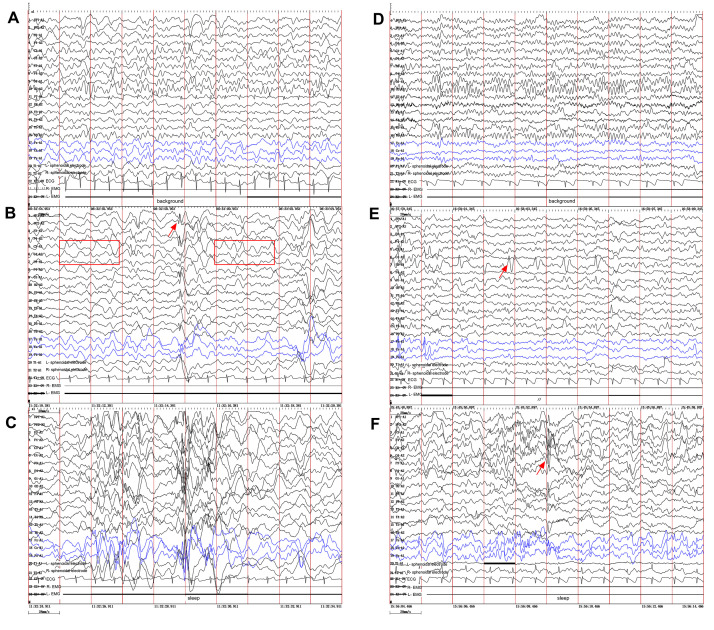
**Video electroencephalography (VEEG) findings in the two brothers**. (A–C) Proband (younger brother). (A) Slowing of the background activity. (B) Abnormal 2–4 Hz slow waves (red box in B) mixed with spikes and spike-slow waves (red arrow), bilateral frontally predominant, especially during sleep (C). (D–F) Elder brother. (D) Normal background rhythm. (E) Frequent focal spikes and sharp waves (red arrow) localized to the left parietal and mid-posterior temporal regions during the awake EEG. (F) Similar epileptiform discharges were observed (red arrow) during sleep EEG. Ipsilateral ear reference montage. EEG, electroencephalogram.

Family history: The parents were healthy and non-consanguineous, with no family history of epilepsy, psychiatric disorders, neurological diseases, or genetic conditions. Both paternal and maternal grandparents were also healthy. Developmental history: The mother was gravida 2 para 2 (G2P2) with pregnancy-induced hypertension. The proband was born full term by vaginal delivery, with no perinatal hypoxia, pathological jaundice, or febrile seizures. No developmental abnormalities were noted in the first year. He walked at 14 months but exhibited delayed speech, only producing single words by age 3, with intellectual development lagging behind peers. His older brother was born full term via vaginal delivery without perinatal complications. His early developmental milestones were within normal limits (walking at 12 months, speaking simple words at 15 months), with essentially normal intellectual development compared to peers. Neurological Examination: The proband exhibits developmental delay. Due to a lack of cooperation, higher cognitive functions could not be thoroughly assessed, although he demonstrated the ability to communicate in simple terms. Fine motor skills were underdeveloped. Muscle strength was grade 5 in all four limbs, with a mild subjective weakness. Tendon reflexes were normal, and no pathological signs were present. Neurological examination of the patient’s elder brother revealed a speech delay. Orientation, memory, calculation, and comprehension were essentially intact. Muscle strength was grade 5 in all limbs, with mild hypotonia. Ancillary investigations, including assessments of thyroid function, lactate levels, endocrinology, and complete blood count, yielded normal results. Both siblings underwent cranial MRI, VEEG, and genetic testing. MRI of the cranium revealed no abnormalities for both the patient and his elder brother. The cerebral hemispheres were symmetrical, the demarcation between gray and white matter was distinct, and no abnormal signals were detected in the brain parenchyma (Fig. 1). The EEG for the younger brother (Fig. 2, left panel) showed a generalized slowing of the background activity. Interictal recordings revealed abnormal 2–4 Hz slow waves mixed with spikes and spike-slow wave complexes, which were bilaterally frontally predominant. In contrast, the elder brother (Fig. 2, right panel) presented with a normal background. His interictal EEG showed frequent focal spikes and sharp waves localized to the left parietal and mid-posterior temporal regions. The Wechsler Intelligence Scale for Children (WISC) assessment of the patient indicated significant cognitive deficits, with a score of 57 points, classified as Extremely Low. In contrast, his elder brother obtained a score of 73 points, which falls within the Borderline range.

**Fig. 3.**
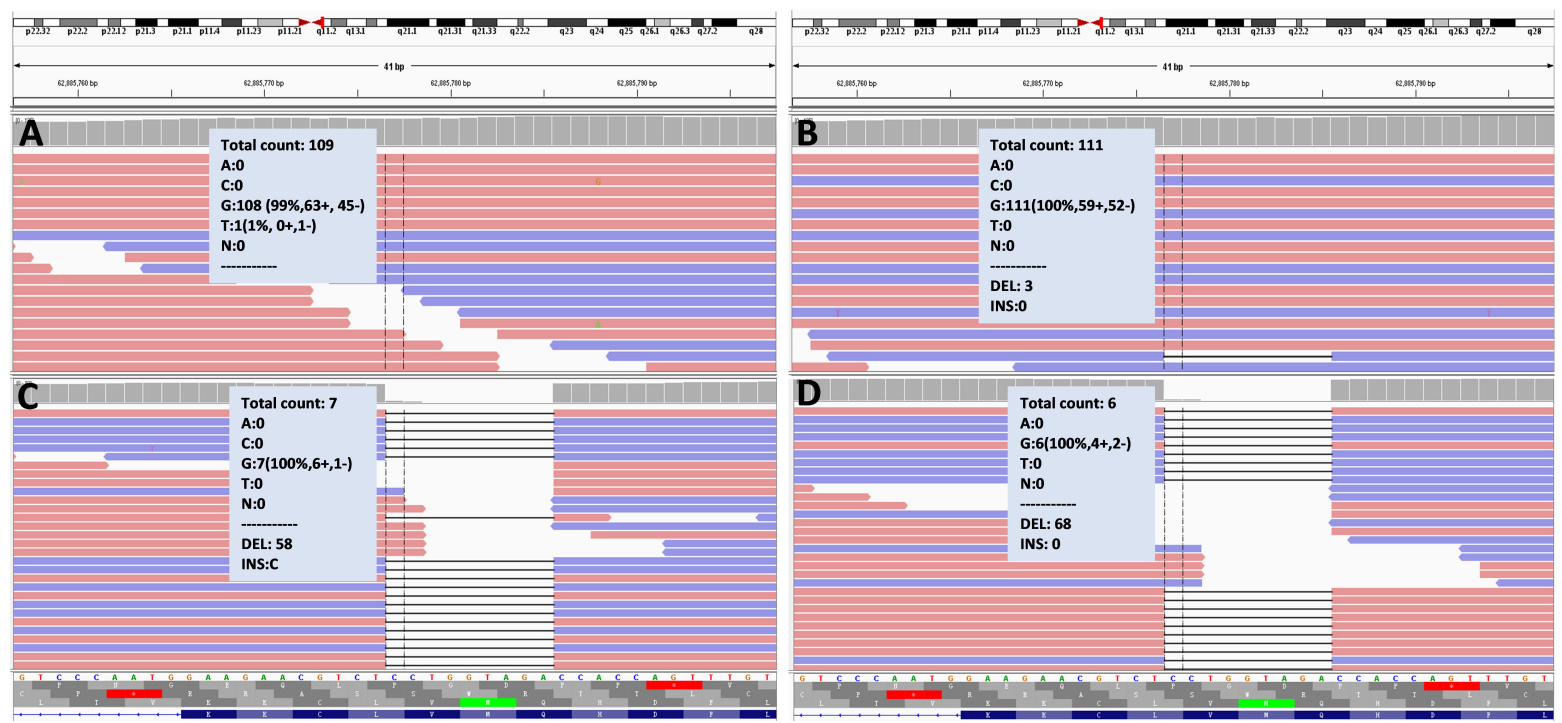
***ARHGEF9* gene analysis in the patient’s family by whole-exome sequencing**. Whole-exome sequencing results: (A) indicates that the patient’s father does not possess the *ARHGEF9* mutation, exhibiting a normal genotype. (B) identifies three deletions in the sample from the patient’s mother. (C,D) reveal that both the patient (C) and his elder brother (D) carry the *ARHGEF9*:c.1037_1045del p.(Gln346_Val348del) deletion. ARHGEF9, Cdc42 Guanine Nucleotide Exchange Factor 9.

Whole-exome sequencing (WES) was performed by a commercial clinical genetic testing laboratory (KingMed Diagnostics, Guangzhou, Guangdong, China). Genomic DNA was isolated from peripheral venous blood samples using a standard commercial kit (cat. no. 51304, QIAGEN, Venlo, Netherlands). WES was performed using the IDT xGen Exome Hyb Panel v2 (Integrated DNA Technologies, Coralville, IA, USA) for targeted exome enrichment, followed by massively parallel sequencing on the NovaSeq 6000 high-throughput sequencing platform (Illumina, San Diego, CA, USA). Sequencing achieved a mean depth of 150× for germline genetic analysis, with at least 98% of the targeted regions covered at a minimum depth of 20× and a base quality score (Q30) exceeding 90%. Raw sequencing reads were aligned to the human reference genome assembly (GRCh38/hg38), and bioinformatic analysis was performed to detect single-nucleotide variants (SNVs) and small insertions and deletions (indels). WES revealed that both the patient and his older brother carried deletion variant in *ARHGEF9* (NM_015185.2): c.1037_1045del, p.(Gln346_Val348del) (Fig. 3). Both siblings were hemizygous for this variant. Sanger sequencing confirmed that neither parent carried the variant at this locus, indicating that the variant was *de novo*. The patient’s mother carried the *ARHGEF9* gene variant at an allele frequency of approximately 2.6%, whereas no variant was detected in the father. Considering the genetic results in both sons, the mother was suspected to be a potential gonadal mosaic carrier.

Treatment: The younger brother was now treated with levetiracetam (1 g twice daily), lamotrigine (150 mg twice daily), topiramate (100 mg twice daily), and perampanel (8 mg nightly). The therapeutic response was suboptimal, as the patient continued to experience intermittent seizures with a frequency of 2 to 5 episodes per month. Due to inadequate management of epileptic symptoms, the patient underwent vagus nerve stimulation (VNS) surgery in 2023. Neurostimulation was activated 2 weeks postoperatively with initial parameters: current 0.25 mA, frequency 30 Hz, pulse width 250 µs, stimulation duration 30 s, and inter-stimulation interval 5 min. The stimulation current was titrated upward by 0.1–0.3 mA every 1–2 weeks based on the patient’s tolerance, and gradually increased to 1.5 mA by 3 months after surgery. Thereafter, parameters were adjusted every 3 months with further escalation of the stimulation current until reaching the maximum tolerable current of 2 mA. The settings were then optimized to a frequency of 50 Hz, pulse width of 500 µs, and inter-stimulation interval of 3 min. Postoperatively, within six months, the seizure frequency decreased to approximately 1 to 3 episodes per month. However, one year post-surgery, the frequency of seizures gradually increased, reverting to 2 to 5 episodes per month, indicating no significant improvement compared to the preoperative state. In contrast, the patient’s elder brother, who was treated with sodium valproate and oxcarbazepine, achieved effective seizure control, experiencing only occasional seizures 1 to 2 times per year. The basic clinical characteristics and treatment of the two brothers are shown in Table 1.

**Table 1.** **The basic clinical characteristics and treatment of the patients**.

	Patient 1 (younger brother)	Patient 2 (elder brother)
Age of onset	11 m	18 m
Clinical symptoms	Epilepsy; severe developmental delay	Epilepsy; moderate developmental delay
Seizure type	Focal onset seizure, Generalized tonic-clonic seizure	Focal onset seizure
MRI	No obvious structural abnormalities	No obvious structural abnormalities
VEEG	Slow wave background, widespread 2–4 Hz slow waves, spikes and spike-wave complexes predominantly in the bilateral anterior regions	Normal background, widespread spikes, spikes slow waves in the left parietal and mid-posterior temporal regions
WISC	57	73
Treatment	LEV, LTG, TPM, PRE, VNS	VPA, OXC
Outcome	Refractory epilepsy, with persistent seizures occurring 2-5 episodes per month	Seizures are largely controlled with treatment, occurring 1–2 times per year

Abbreviations: WISC, Wechsler Intelligence Scale for Children; LEV, levetiracetam; LTG, lamotrigine; TPM, topiramate; PRE, perampanel; VNS, vagus nerve stimulation; VPA, valproic acid; OXC, oxcarbazepine; m, month.

To date, more than 40 children with *ARHGEF9* gene variants have been reported in the literature. Their clinical phenotypes include developmental delay, epilepsy, hyperarousal to noise, hyperactivity, hypotonia, epileptic encephalopathy, autism spectrum disorder, and dysmorphic features. MRI reveals cerebral cortical and cerebellar vermis atrophy, corpus callosum hypoplasia, malformations, etc. [2, 3, 4, 5, 6, 7, 8, 9, 10]. Representative cases reported in the literature are summarized in Table 2 (Ref. [3, 4, 5, 6, 7]).

**Table 2.** **Genotype and phenotype analysis of the *ARHGEF9* gene reported in literature**.

Reference	Mutation	Inheritance	Sex (n)	Age of onset	Clinical feature	Effective treatment
Lesca *et al*. [3]	Xq11.11 deletion:arrXq11.1(61848414-63138698)	*De novo*	Male (1)	6 y	Developmental delay; epilepsy, macrosomia; dysmorphic features	OXC, LEV
Freri *et al*. [4]	p.G496L	*De novo*	Male (1)	16 y	Epilepsy; intellectual disability	Refractory
Bhat *et al*. [5]	Xq11.1-Xq11.2 deletion:arrXq11.1-Xq11.2(62970571-63052696)	*De novo*	Female (1)	8 y	Autism spectrum disorder	N/A
Wang *et al*. [6]	p.R290C	*De novo*	Male (4)	10 y (median)	Intellectual disability; epileptic encephalopathy	Refractory
Yang *et al*. [7]	NM_015185.2:exon8: c.1094G>A (p.R365H)	Maternal	Male (1)	4 y	Epilepsy; severe developmental delay	VPA
	NM_015185.2:exon8: c.1162A>G (p.M388V)	Maternal	Male (1)	10 y	Epilepsy; hyperarousal to noise; severe developmental delay	Refractory
	NM_015185.3:exon8:c.1094G>A (p.R365H)	Maternal	Male (1)	3 y 7 m	Recurrent febrile seizures; epilepsy; severe developmental delay	LEV
	NM_001173479.1:exon5: c.639C>G (p.D213E)	*De novo*	Male (1)	2 y 9 m	Epilepsy; moderate developmental delay	LEV
	NM_001173479:exon2: c.188G>A (p.R63H)	*De novo*	Male (1)	2 y 4 m	Epilepsy; mild developmental delay	VPA

Abbreviations: N/A, Not available; y, year.

## 3. Discussion

The patients presented with early-onset epileptic seizures and developmental delay as the primary clinical manifestations. Ancillary investigations revealed no evidence of infection, while biochemical and urinary metabolic assessments did not indicate a metabolic disorder. Furthermore, cranial MRI excluded the presence of structural abnormalities. In conjunction with genetic testing, these findings are indicative of developmental and epileptic encephalopathy associated with the *ARHGEF9* gene [7, 11]. Based on the early onset of epilepsy, global developmental delay/intellectual disability, abnormal VEEG findings, and the identified pathogenic *ARHGEF9* gene variant, the clinical presentations of the two children meet the diagnostic criteria for DEE8.

The *ARHGEF9* gene is located at Xq11.1 and is ubiquitously expressed across tissues, with predominant expression in brain tissue [12]. It is expressed in most neurons in the cornu ammonis area 1 (CA1), area 3 (CA3) and dentate gyrus regions of the hippocampus throughout development. *ARHGEF9* encodes collybistin, a protein that is essential for the gephyrin-dependent postsynaptic clustering of glycine and γ‑aminobutyric acid A (GABAA) receptors [2]. Based on the documented cases of *ARHGEF9* gene variants, the clinical phenotype may include seizures that may present as early as the neonatal period, with a variety of seizure types, including focal or generalized tonic, myoclonic, and tonic-clonic seizures [6, 8]. EEG findings may demonstrate generalized, bilateral, multifocal, or unifocal epileptiform discharges. In addition to epilepsy, clinical manifestations may include intellectual disability, sensory hypersensitivity, sleep disorders, hyperekplexia, hyperactivity, impulsivity, and autism spectrum disorder. While most cranial imaging studies do not show significant abnormalities, a minority of children may exhibit findings on MRI, such as frontal lobe hypoplasia, polymicrogyria, or cerebral atrophy [4, 9, 10].

Through WES analysis, we identified a hemizygous *ARHGEF9* gene variant, specifically c.1037_1045del. This particular deletion variant has not been previously documented in the literature. The mother was found to carry the *ARHGEF9* gene mutation at a low frequency (~2.6%), which was considerably lower than the expected 50% in a typical heterozygous carrier. This finding suggests that the mutation predominantly exists in ovarian/germ cells rather than in somatic cells throughout the body, consistent with the possibility of gonadal mosaicism. Direct analysis of germ cell-related samples would provide the most accurate estimation of the mosaic ratio; however, such samples are difficult to obtain in clinical practice and were not analyzed in the present case. The patient inherited this mutated X chromosome from his mother. Because males possess only one X chromosome (hemizygous), they fully manifest the phenotypic effects of the mutation, resulting in symptoms such as epilepsy and cognitive impairment. The patient experienced recurrent seizures despite treatment with multiple anti-epileptic drugs and VNS therapy. In contrast, his older brother, who demonstrated better cognitive function, achieved good seizure control with two anti-epileptic drugs. The two affected brothers carried the same *ARHGEF9* deletion variant but showed distinct phenotypic manifestations, and the underlying mechanism remains to be fully elucidated. Several factors may account for this phenotypic variability. First, genetic modifiers and differences in genomic background could modulate disease expression despite sharing an identical pathogenic variant. Second, earlier seizure onset (11 vs. 18 months) and different initial treatment responses in the proband may have resulted in a higher epileptiform load during early brain development, which could exert more severe impacts on maturing neural networks and thus contribute to poorer neurodevelopmental outcomes. In addition, environmental and educational factors, such as the intensity of early intervention, rehabilitation training, and family support, may also modify long-term neurocognitive prognosis.

This study presents the clinical characteristics and genetic findings of two patients with developmental and epileptic encephalopathy caused by a deletion variant in the *ARHGEF9* gene, thereby broadening the known variant spectrum of this gene. Early genetic testing can facilitate a definitive diagnosis and prognosis assessment, providing an important basis for genetic counseling and prenatal diagnosis. However, this study has several limitations: no functional assays were conducted to directly verify the effect of the identified novel deletion on collybistin function, and the relatively short duration of neuropsychological follow-up may incompletely reflect the longitudinal developmental trajectory. Therefore, further studies involving larger patient cohorts and functional experiments are required to better elucidate the genotype-phenotype correlations in *ARHGEF9*-related disorders.

## Availability of Data and Materials

The datasets used and analyzed during the present study are available from the corresponding author on reasonable request.

## Supplementary Material

Supplementary material associated with this article can be found, in the online version, at https://doi.org/10.31083/RN46598.
